# LASSO Regression with Multiple Imputations for the Selection of Key Variables Affecting the Fatty Acid Profile of *Nannochloropsis oculata*

**DOI:** 10.3390/md21090483

**Published:** 2023-09-02

**Authors:** Vasilis Andriopoulos, Michael Kornaros

**Affiliations:** 1 Laboratory of Biochemical Engineering & Environmental Technology (LBEET), Department of Chemical Engineering, University of Patras, 26504 Patras, Greece; billandri@upatras.gr; 2 Institute of Circular Economy and Environment (ICEE), University of Patras’ Research and Development Center, 26504 Patras, Greece

**Keywords:** de novo synthesis, heavy metals, lipid induction, nitrogen starvation, oxygen, parametric methods, phosphate starvation, polar lipids, statistical analysis, TAG

## Abstract

The marine microalga *Nannochloropsis oculata* has garnered significant interest as a potential source of lipids, both for biofuel and nutrition, containing significant amounts of C16:0, C16:1, and C20:5, n-3 (EPA) fatty acids (FA). Growth parameters such as temperature, pH, light intensity, and nutrient availability play a crucial role in the fatty acid profile of microalgae, with *N. oculata* being no exception. This study aims to identify key variables for the FA profile of *N. oculata* grown autotrophically. To that end, the most relevant literature data were gathered and combined with our previous work as well as with novel experimental data, with 121 observations in total. The examined variables were the percentages of C14:0, C16:0, C16:1, C18:1, C18:2, and C20:5, n-3 in total FAs, their respective ratios to C16:0, and the respective content of biomass in those fatty acids in terms of ash free dry weight. Many potential predictor variables were collected, while dummy variables were introduced to account for bias in the measured variables originating from different authors as well as for other parameters. The method of multiple imputations was chosen to handle missing data, with limits based on the literature and model-based estimation, such as using the software PHREEQC and residual modelling for the estimation of pH. To eliminate unimportant predictor variables, LASSO (Least Absolute Shrinkage and Selection Operator) regression analysis with a novel definition of optimal lambda was employed. LASSO regression identified the most relevant predictors while minimizing the risk of overfitting the model. Subsequently, stepwise linear regression with interaction terms was used to further study the effects of the selected predictors. After two rounds of regression, sparse refined models were acquired, and their coefficients were evaluated based on significance. Our analysis confirms well-known effects, such as that of temperature, and it uncovers novel unreported effects of aeration, calcium, magnesium, and manganese. Of special interest is the negative effect of aeration on polyunsaturated fatty acids (PUFAs), which is possibly related to the enzymatic kinetics of fatty acid desaturation under increased oxygen concentration. These findings contribute to the optimization of the fatty acid profile of *N. oculata* for different purposes, such as production of, high in PUFAs, food or feed, or production of, high in saturated and monounsaturated FA methyl esters (FAME), biofuels.

## 1. Introduction

*Nannochloropsis oculata* has emerged as a promising microalga for the production of high-value biomolecules, particularly long-chain polyunsaturated fatty acids (LC-PUFA), such as C20:5, n-3 or eicosapentaenoic acid (EPA) [1,2]. EPA, as a key component of the fatty acid (FA) profile, plays a vital role in human health and nutrition, offering benefits such as cognitive enhancement, mental health improvement, and cardiovascular protection [3]. Additionally, under stressful conditions, such as nitrogen limitation, *N. oculata* can accumulate large amounts of saturated FAs, primarily C16:0 or palmitic acid, and monounsaturated FAs like C16:1 or palmitoleic acid, both of which are suitable for biodiesel production [4]. Therefore, adjustment of the cultivation conditions according to the end goal is critical for industrial scale production.

The FA composition of *N. oculata* can be affected by various factors, including temperature, light intensity, pH, and the type of reactor used for cultivation [1,5,6]. Temperature plays a significant role in the FA composition of *N. oculata*, with total lipid content increasing with temperature, while polyunsaturated fatty acid (PUFA) levels decrease and saturated fatty acid (SFA) levels increase [5]. Optimal growth and total lipid productivity for *N. oculata* have been reported at 20–25 °C, with the highest EPA production achieved between 14–20 °C [5,7].

Light intensity is another factor that can influence the FA profile of *N. oculata*. Su et al. reported that irradiance of 500 μmol photons m^−2^ s^−1^ resulted in the highest total lipid productivity [8]. In another study, Martínez-Macias et al. found that saturating light intensity reduced the levels of SFAs and increased the combined levels of monounsaturated fatty acids (MUFAs) and PUFAs, although the variation was marginal [6]. Additionally, the type of reactor used for cultivation had a great impact on the FA profile, as demonstrated by the same authors, who observed a decrease in PUFAs (20:5n-3) and (20:4n-3) when using a tubular reactor instead of an Erlenmeyer flask [6].

The pH and light period during cultivation have also been shown to impact the FA composition and antioxidant capacity of *N. oculata* [1]. The study revealed that optimal conditions for productivity and biomass composition varied, and that further investigation is necessary to better understand the effects of these variables on the FA profile. Additionally, a plethora of other factors including metal concentration [9], CO_2_ levels [10], agitation [11], light color [12], and magnetic fields [13] have a demonstrated effect on the fatty acid content of *Nannochloropsis* and other microalgae. Evaluating the effects of these factors on the FA composition of *N. oculata* is crucial for optimizing its cultivation conditions and maximizing its potential as a source of biofuels and high-value biomolecules.

In the era of big data, the challenge of variable selection from large numbers of variables, often exceeding the number of observations, is a pressing issue in many scientific fields. Parametric methods, such as the Least Absolute Shrinkage and Selection Operator (LASSO) regression, have emerged as powerful tools to address this challenge [14]. LASSO regression, a type of linear regression that uses shrinkage, is particularly adept at handling high-dimensional data, as it performs both variable selection and regularization to enhance the prediction accuracy and the interpretability of the statistical model it produces. The LASSO algorithm’s underlying principle revolves around striking a balance between the complexity of the model and its predictive capability. By adding a penalty term proportional to the absolute values of the regression coefficients, LASSO effectively encourages the reduction of less impactful coefficients to exactly zero. This property not only aids in selecting relevant variables but also guards against overfitting, a common concern in situations where the number of variables surpasses the available observations. 

The versatility of LASSO finds application in a multitude of scientific domains. In the realm of genomics, LASSO aids in discerning the genetic markers that are truly relevant to certain traits or diseases from the noise in high-throughput genetic data [15]. In economics, it assists in untangling the intricate relationships between numerous economic indicators, contributing to more accurate predictive models [16]. Additionally, LASSO’s utility extends to fields like climate science, where it aids in identifying the pivotal climatic variables driving specific environmental phenomena [17].

However, real-world datasets often contain missing values, which can introduce bias, reduce the efficiency of the statistical analyses, and lead to a loss of information. Multiple imputations, a flexible and widely applicable type of approach, have been proposed to handle missing data [18]. This method replaces each missing value with a set of plausible values to generate multiple complete datasets. The variability between these imputations reflects the uncertainty about the right value to impute. Pooling methods such as Rubin’s rule are then used to combine the results obtained from each of these datasets, providing a single estimate and confidence interval that incorporate both within-imputation and between-imputation variability, thus yielding valid statistical inferences that properly reflect the uncertainty due to missing values [18].

The goal of the current study is to identify the key growth variables influencing the FA profile of *N. oculata* using data obtained from different experiments conducted under various conditions and reactor types, as well as the relevant literature data. To comprehensively analyze the relationships between growth variables and the FA profile of *N. oculata*, we employed LASSO regression analysis and stepwise linear regression, inspired by the recently developed High Dimensional Selection with Interactions (HDSI) algorithm, which aims to incorporate interaction terms in LASSO regression [19]. Stepwise linear regression is a computationally intensive but powerful method for creating sparse models. The combination of LASSO regression with stepwise regression should provide a thorough examination of the complex relationships between growth variables and the FA profile of *N. oculata*, setting the stage for future optimization efforts. By employing this specific combination of advanced regression techniques, we aim to provide valuable insights into the modulation of *N. oculata* FA profile for different purposes, such as the production of high-value PUFA-rich biomass, suitable for food or feed, or rich in saturated and monounsaturated FAs biomass, with a lipid profile suitable for biofuels.

## 2. Results

**Figure 1:**
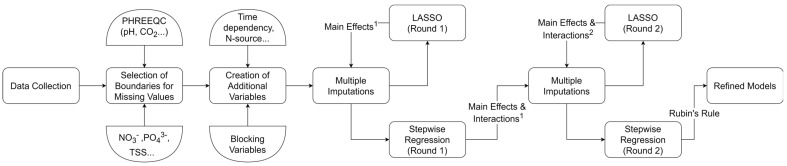
Methodology followed during the analysis presented in this article.

The methodology followed is presented in Figure 1 and extensively discussed in the Materials and Methods section. Briefly, original data were combined with data from the literature. Additional variables were added to account for data origin, nitrogen source, and the temporal profile of selected variables. Upper and lower limits were chosen for missing data using in-house developed models and educated assumptions. LASSO analysis with multiple imputations for the missing data was performed to reduce the variables to only a fraction of their initial number (115) in order to assist the subsequent selection of significant interactions with stepwise linear regression. In total, two rounds of LASSO regression and two rounds of stepwise regression were performed. Refined sparse models were finally acquired with pooling of multiple coefficients with Rubin’s rule. A full list of the variables included in the analysis, with their descriptions and coded names, is provided in the Supplementary Materials (S1).

### 2.1. First Round of LASSO Regression

The results of the first round of LASSO regression (Table 1, Table 2 and Table 3) are presented in the paragraphs below.

For the percentage of C14:0 in total FAs (C14:0) (Table 1), its ratio to C16:0 (C14:0/C16:0), and its percentage in the biomass in terms of ash free dry weight (AFDW) (C14:0%AFDW), the selected variables were potassium concentration (cK), phosphate concentration (cPO4), CO_2_ partial pressure (pCO2), 4-day average CO_2_ partial pressure (pCO2_4DaysAv), temperature (T), 2-day average aeration rate in volumes of air per working volume per minute or vvm (VVM_2DaysAv), 4-day average dissolved CO_2_ concentration (CO2aq_4DaysAv), light intensity (LI), and 4-day average photon flux per volume (LV_4DaysAv). All of these variables had a positive effect, except for VVM_2DayAv and LV_4DaysAv.

For the percentage of C16:0 in total FAs (C16:0) and its percentage in the biomass in terms of ash free dry weight (C16:0%AFDW), the selected variables were boron concentration (cBOH3), cobalt concentration (cCo), cK, nitrate as a nitrogen source (N-source NO3), 3-day average nitrate concentration (cNO3_3DaysAv), 2-day average urea concentration (cUrea_2DaysAv), sodium concentration (cNa), 4-day average phosphate concentration (cPO4_4DaysAv), sulfate concentration (cSO4), 3-day average CO_2_ partial pressure (pCO2_3DaysAv), T, 3-day average temperature (T_3DaysAv), the presence of aeration (Aeration), VVM_2DaysAv, pH (pH), 4-day average pH (pH_4_DaysAv), CO2aq_4DaysAv, LI, light period (LP), and 4-day average biomass concentration in terms of ash free dry weight (DW_4DaysAv). cBOH3, cCo, NO3_3DaysAv, Urea_2DaysAv, cNa, cPO4_4DaysAv, cSO4 andVVM_2DaysAv had a negative effect, while the rest of the variables displayed a positive effect.

For the percentage of C16:1 in total FAs (C16:1), its ratio to C16:0 (C16:1/C16:0), and its percentage in the biomass in terms of ash free dry weight (C16:1%AFDW), the selected variables were iron concentration (cFe), cK, manganese concentration (cMn), magnesium concentration (cMg), cNO3_3DaysAv, 4-day total nitrogen concentration (totalN_4DaysAv), cPO4_4DaysAv, cSO4, silicon concentration (cSi), pCO2_4DaysAv, T, T_3DaysAv, Aeration, pH_4_DaysAv, CO2aq_4DaysAv, 2-day average dissolved bicarbonate concentration (HCO3_2DaysAv), LI, photon flux per volume (LV), LP, and DW_4DaysAv. All of these variables had a positive effect, except for cK, T_3DaysAv and HCO3_2DaysAv.

For the percentage of C18:1 in total FAs (C18:1), its ratio to C16:0 (C18:1/C16:0), and its percentage in the biomass in terms of ash free dry weight (C18:1%AFDW), the selected variables were copper concentration (cCu), cFe, cMn, 2-day average nitrate concentration (cNO3_2DaysAv), 4-day average nitrate concentration (cNO3_4DaysAv), Urea_2DaysAv, 3-day average sodium concentration (cNa_3DaysAv), 4-day average phosphate concentration (cPO4_4DaysAv), 2-day average CO_2_ partial pressure (pCO2_2DaysAv), Aeration, 2-day average dissolved CO_2_ concentration (CO2aq_2DaysAv), LI, 3-day average photon flux per volume (LV_3DaysAv), LV_4DaysAv, LP, and DW_4DaysAv. cFe, NO3_2DasAv, NO3_4DasAv, Urea_2DaysAv, and cPO4_dDaysAv had a negative effect, while the rest of the variables displayed a positive effect.

For the percentage of C18:2 1 in total FAs (C18:2), its ratio to C16:0 (C18:2/C16:0), and its percentage in the biomass in terms of ash free dry weight (C18:2%AFDW), the selected variables were calcium concentration (cCa), cCu, cMn, NsourceNO3, urea as a nitrogen source (NsourceUrea), nitrate concentration (cNO3), 4-day average urea concentration (cUrea_4DaysAv), cPO4_4DaysAv, 4-day average temperature (T_4DaysAv), Aeration, 2-day average pH (pH_2DaysAv), LV, LV_4DaysAv, and 2-day average biomass concentration average biomass concentration in terms of ash free dry weight (DW_2DaysAv). All of these variables had a positive effect, except for cCa, NsourceNO3, Aeration, and pH_2DaysAv, which had a negative effect.

For the percentage of C20:5, n-3 in total FAs (C20:5n3), its ratio to C16:0 (C20:5n3/C16:0), and its percentage in the biomass in terms of ash free dry weight (C20:5n3%AFDW), the selected variables were cBOH3, cNO3_3DaysAv, 4-day average sodium concentration (cNa_4DaysAv), cPO4_4DaysAv, cSi, salinity (S), T, Aeration, CO2aq_4DaysAv, LI, and LP. T, Aeration, CO2aq_4DaysAv, LI, and LP had a negative effect, while the rest of the variables showed a positive effect.

In summary, the 1^st^ round of LASSO regression removed most of the initial variables, with the remaining variables being related to well-established growth parameters, such as temperature, pH, CO_2_ partial pressure, light intensity or nutrient concentration or less studied parameters such as metal concentration and light period. One variable that emerged as important is the presence of aeration, which exerted a negative effect on both C18:2 and C20:5, n-3. The full dataset of results of the 1^st^ LASSO regression round is provided in the Supplementary Materials (S2).

### 2.2. First Round of Stepwise Regression

During the first round of stepwise regression, the variables that were selected and presented above were fitted to linear models with interactions in a stepwise manner. The algorithm starts from a model including all the main effects and interactions and excludes or adds terms one at a time. As the process continues, only the most significant terms remain. It is a computationally intensive process, but it is a powerful method that can handle multicollinearity better than simple linear regression and was thus preferred. It is also similar to the bootstrapping method used by Jain and Xu in their HDSI algorithm [19]. The first round of stepwise regression was only an intermediate stage, therefore its results are provided in the Supplementary Materials (S3).

### 2.3. 2^nd^ Rounds of LASSO and Stepwise Regression

The second round of LASSO regression included the main effects and interactions present in the models resulting from the first round of stepwise regression. This resulted in a reduced number of main effects and interactions. The main effects that were present in the models, either individually or as part of an interaction, were included in the second and final round of stepwise regression. However, only the most notable interactions (as described in the Materials and Methods section) were included to prevent overfitting. The second round of stepwise regression further reduced the number of terms resulting in sparse interpretable models. Only the results for the final models (Table 4, Table 5, Table 6, Table 7, Table 8 and Table 9) are presented below (and the full dataset in Supplementary Materials S3), while the results of the second round of LASSO are provided in the Supplementary Materials (S2).

C14:0 was notably influenced by T, exhibiting a positive effect (mean *p*-value < 0.05). A secondary factor (trimmed *p*-value < 0.05) was the positive interaction of T with cPO4. cPO4 also contributed to the model with a positive coefficient, albeit with a *p*-value exceeding 0.05.

Conversely, cPO4 was absent from the model for C14:0/C16:0, but its interaction with cK was notable (*p*-value < 0.05) with a positive coefficient. The only other term in this model, besides the intercept, was cK, which also had a positive coefficient.

The model for C14:0%AFDW was primarily influenced by variables related to aeration rate and CO_2_ supply. Both pCO2_4DaysAv and VVM_2DaysAv exhibited notable negative effects (mean *p*-value < 0.05), while their interaction had a notable positive effect. CO2aq_4DaysAv and its interaction with pCO2_4DaysAv also had a negative effect (*p*-value >> 0.05).

Parameters with positive mean coefficients in the model for C16:0 included cK, NsourceNO3 and its interaction with T_3DaysAv, NO3_3DaysAv, Aeration, LP, the interactions between pH and both T (*p*-value < 0.05) and LI, as well as the interactions of Aeration with both LI and LP (trimmed mean *p*-value < 0.05). Negative influences were observed for cNa, cPO4_3DaysAv, VVM_2DaysAv (trimmed mean *p*-value < 0.05), LI, DW_4DaysAv, and the interactions of NO3_3DaysAv with both cNa (trimmed mean *p*-value < 0.05) and VVM_2DaysAv.

For C16:0%AFDW, the positive effect of cK, the negative effect of cPO4_3DaysAv, the negative effect of VVM_2DaysAv, and the positive effect of the interaction between Aeration and LI were notable (mean *p*-value < 0.05). A secondary factor (trimmed mean *p*-value < 0.05) was the positive effect of the interaction between pCO2_3DaysAv and VVM_2DaysAv.

The model for C16:1 contained, apart from an intercept, only NO3_3DaysAv and LV, both of which had a negative effect (*p* >> 0.05), as well as their interaction, which had a notable negative effect (mean *p*-value < 0.05).

Conversely, the model for C16:1/C16:0 contained the largest number of terms from all models presented in this article, the most notable of which were the negative interaction between cMg and LV (*p*-value < 0.05), the interaction between T and pH_4DaysAv (trimmed mean *p*-value < 0.05), which presented a negative effect, and cSi (trimmed mean *p*-value < 0.05), which had a positive effect. Other terms with a positive effect (*p* > 0.05) were cMn and cMg, cPO4_4DaysAv and its interaction with NO3_3DaysAv, pCO2_4DaysAv, T_3DaysAv and its interaction with pH_4DaysAv, pH_4DaysAv, HCO3_2DaysAv and the interaction between cFe and NO3_3DaysAv. The remaining terms had a negative effect (*p* > 0.05) and included totalN_4DaysAv as well as its interactions with both HCO3_2DaysAv and LV, T and its interaction with pH_4DaysAv, CO2aq_4DaysAv and the interaction between Aeration and LP.

C16:1%AFDW was influenced by many of the same parameters as C16:1/C16:0, like cMg and cPO4_4DaysAv, which, in that case, had negative effects (*p*-value > 0.05). cSi also appears with an opposite effect (negative), which is, akin to the case of C16:1/C16:0, moderately important (trimmed mean *p*-value < 0.05). NO3_3DaysAv and its interaction with the pH_4DaysAv presented moderately significant effects (trimmed mean *p*-value < 0.05), negative and positive respectively. pH_4DaysAv had a notable negative effect (mean *p*-value < 0.05), while the interactions between cSi and LI, and between pCO2_4DaysAv and DW_4DaysAv had strong positive effects (mean *p*-value < 0.05). LI and the DW_4DaysAv showed negative effects (*p*-value > 0.05).

The most important (mean *p*-value < 0.05) terms for C18:1 were NO3_3DaysAv, pCO2_2DaysAv, and the interaction between the Aeration and LP, all of which had a positive effect. The interaction between Aeration and LV_4DaysAv also had a positive effect (trimmed mean *p*-value < 0.05), while CO2aq_2DaysAv had a negative effect (trimmed mean *p*-value < 0.05). Other negative influences (*p*-value > 0.05) were those of NO3_2DaysAv and NO3_3DaysAv, CO2aq_2DaysAv, LI, and LV_3DaysAv. The remaining terms had positive effects (*p*-value > 0.05) and included cPO4_4DaysAv, DW_4DaysAv and the interaction between can_3DaysAve and LV_3DaysAv.

Aeration dominated the model of C18:1/C16:0 with its positive interactions (mean *p*-value < 0.05) with both LV_4DaysAv and LP, while cNa_3DaysAv also had a positive effect (trimmed mean *p*-value < 0.05). The interaction between cNa_3DaysAv and LP had a positive effect (*p*-value > 0.05), while negative influences (*p*-value > 0.05) were observed for CO2aq_2DaysAv and the interactions of cNO3_2DaysAv and cNO3_4DaysAv with LI. 

Aeration was also important for C18:1%AFDW with a notable positive effect (mean *p*-value < 0.05), while the interaction of pCO2_2DaysAv with LV_3DaysAv was also notable. The interaction between LV_4DaysAv and DW_4DaysAv was moderately important (trimmed mean *p*-value < 0.05) with a positive effect. Other terms included in the model were cNO3_2DaysAv and cNO3_4DaysAv, both with a negative effect, LV_4DaysAv and DW_4DaysAv (both with negative effect), pCO2_3DaysAv (positive effect), CO2aq_2DaysAv (negative effect), LI (positive effect), and the positive interactions of DW_4DaysAv with both LV_3DaysAv and LV_4DaysAv.

The model of C18:2 was primarily influenced by cCa, Aeration, and the interaction between cMn and T_4DaysAv, with the first two having a significant (mean *p*-value << 0.05) negative effect and the third showing a positive effect (mean *p*-value << 0.05). cPO4_4DaysAv was also included in the model and had a positive effect (*p*-value > 0.05).

The negative effects of cCa (mean *p*-value << 0.05) and the interaction between the Aeration and pH_2DaysAv (mean *p*-value < 0.05), as well as the positive effect of cNO3 (mean *p*-value << 0.05), were the most important effects in the model for C18:2/C16:0, while cMn had a moderately important positive effect (trimmed mean *p*-value < 0.05). cPO4_4DaysAv and the interaction between cMn and T_4DaysAv had positive effects, while Aeration had a negative effect, all with *p*-value larger than 0.05.

The interactions between cMn and T_4DaysAv (mean *p*-value << 0.05) and between LV and DW_2DaysAv (mean *p*-value < 0.05), both of which were positive, had the most notable effects on C18:2%AFDW. DW_2DaysAv was included in the model with a moderately positive effect (trimmed mean *p*-value < 0.05), while its interactions with both LV_4DaysAv and cMn also had a positive effect (*p*-value > 0.05). LV, LV_4DaysAv and the interaction between cCa and pH2DaysAv had negative effects (*p*-value > 0.05). 

Similar to the case of C18:2, Aeration was a notable term for C20:5n3, with a negative effect (mean *p*-value < 0.05). The other notable term was T, also with a negative effect. LP had a moderately important negative effect (trimmed mean *p*-value < 0.05), while the interaction between NO3_3DaysAv and S had a positive effect (trimmed mean *p*-value < 0.05). NO3_3DaysAv and S had positive effects (*p*-value > 0.05), while cNa_4DaysAv, LP and its interaction with the T, and the interaction between Aeration and LI all had negative effects (*p*-value > 0.05).

Aeration and T had the most notable effects on C20:5n3/C16:0, both negative (mean *p*-value < 0.05), similarly to C20:5n3, while LP and the interaction between NO3_3DaysAv and cNa_4DaysAv had moderately important negative effects (trimmed mean *p*-value < 0.05). cBOH3, the interaction between NO3_3DaysAv and S, and the interaction between T and LP all had positive effects (*p*-value > 0.05). On the other hand, NO3_3DaysAv and the interactions of Aeration with LI and LP all had negative effects (*p*-value > 0.05).

The second rounds of LASSO and stepwise regression, which included the main effects and interactions derived from the initial models, yielded succinct and interpretable results. Aeration emerged as a pivotal factor consistently influencing the fatty acid composition across various species. It exhibited diverse effects, forming positive interactions with specific variables, such as LV_4DaysAv and LP, while also displaying negative interactions with others. This underscores the importance of aeration control in manipulating fatty acid profiles.

Mineral ions, notably calcium, magnesium, and potassium, played a discernible role in determining fatty acid composition. Their effects were evident through main effects as well as interactions, further highlighting their significance in lipid metabolism regulation. Temperature exhibited significant interactions with several parameters, often leading to shifts in fatty acid profiles. This suggests that temperature management could be a valuable strategy for manipulating lipid production in *N. oculata*.

Nitrogen, especially nitrate, emerged as an influential factor affecting fatty acid profiles. Its interactions with other variables, such as LV and pH_4DaysAv, demonstrated the intricate involvement of nitrogen in lipid synthesis pathways. Additionally, CO_2_-related variables contributed to the models, indicating the relevance of CO_2_ supply in lipid metabolism. The presence of both positive and negative effects underscores the complexity of CO_2_’s role in fatty acid production.

In conclusion, the refined models resulting from the second rounds of LASSO and stepwise regression emphasized the consistent significance of aeration, ion concentrations, temperature, nitrogen sources, and CO_2_-related variables in shaping the fatty acid composition of *N. oculata*. These findings provide valuable insights into the potential manipulation of lipid profiles for various applications, from biodiesel production to nutritional supplementation. The ensuing discussion will delve into the mechanistic underpinnings of these observed effects, connecting them to broader metabolic pathways and potential implications for bioprocess optimization.

## 3. Discussion

In the results section, coded names were used for the variables studied in this article. For the ease of readers focusing on the discussion, variables in this section will be addressed with their original names.

### 3.1. Multi-Level Effects of Temperature

Temperature had a positive effect on C14:0 and C16:0, with increasing temperatures and phosphate concentrations acting synergistically to increase the percentage of C14:0 in total FAs, while temperature seems to enhance the positive effect of increasing pH on C16:0. On the other hand, C16:1 was influenced by temperature in a non-trivial way, with some temperature related terms having a positive effect and others negative. The most significant of them, however, the interaction between temperature and the 4-day average pH, had a negative coefficient. On the other hand, there is a definitive strong negative effect of temperature on C20:5, n-3, both in terms of its percentage in total FAs and its ratio to C16:0. The positive effect of temperature on the accumulation of saturated FAs and its negative effect on PUFAs is well documented in *Nannochloropsis* [1,7,20], while exceptions exist [20]. However, a few remarks on the specific effects of temperature and its interplay with pH and CO_2_ concentration can still be made. 

K1 and K2 of the CO_2_ equilibrium increase with temperature and salinity [21,22,23]. On the other hand, Henry constant for CO_2_ decreases with temperature, which limits CO_2_ transfer from the supplied air to the liquid, while salinity also has a negative but less pronounced effect [24,25]. As K1 and K2 increase, the CO_2_ equilibrium shifts towards bicarbonate and carbonate, respectively, for a given value of pH. Conversely, for given values of K1 and K2, an increase in pH (and therefore a decrease in proton concentration) will shift the CO_2_ equilibrium towards bicarbonate or carbonate, respectively. Microalgae like *Nannochloropsis* utilize carbon concentrating mechanisms, which equip the enzyme carbonic anhydrase, one of the fastest enzymes in nature, to rapidly convert bicarbonate to CO_2_ upon demand in the chloroplast [26]. Thus, for saturated FAs accumulation, conditions favoring the shift of the equilibrium towards bicarbonate, such as high temperature (for example above 30 °C) and moderate pH (for example between 7 and 8.5), might be more important than those promoting the initial dissolution of CO_2_ in water. On the other hand, increasing temperature might disproportionately increase the respiration rate of the cells comparatively to photosynthesis, and thus negatively impact total biomass and total lipid productivity [26]. Therefore, increasing light availability would be beneficial at high temperatures to increase the photosynthetic rate. No interaction was observed between temperature and light supply in any case. There were, however, positive interactions between the presence of aeration or CO_2_ partial pressure and light intensity, light period, or the light flux per volume in the cases of C16:0 and C18:1. That highlights the importance of light under increased carbon supply for the accumulation of saturated and monosaturated FAs. 

### 3.2. Aeration Has a Negative Effect on PUFAs

On the other hand, the interaction between aeration and light intensity or light period was negative for C20:5, n-3. Most importantly, the effect of aeration was significantly negative for both the C20:5, n-3 percentage in total FAs and its ratio to C16:0, while the presence of aeration also had a strong negative effect on C18:2. That could simply reflect the disproportionate accumulation of saturated or monosaturated FAs under stress conditions and increased carbon and light availability compared to the synthesis of PUFAs. Another possibility however could be that the absence of aeration itself has a positive effect on the biosynthesis of PUFAs. A mechanistic explanation for that scenario could be related to the cascade of enzymes involved in PUFA synthesis. The desaturation of carbon bonds for the synthesis of unsaturated FAs is catalyzed by desaturases, enzymes that require molecular oxygen [27,28]. The more double bonds, the more desaturation steps are involved, and thus the more oxygen is required. Therefore, if desaturation is upregulated from increased oxygen concentration, which would be the case in a non-aerated autotrophic microalgal culture, its effect would be more important as the number of double bonds increase, which is compatible with the results presented in this article, with the effect of aeration being positive for saturated FAs, positive or slightly negative for C16:1 and C18:1 and significantly negative for C18:2 and C20:5, n-3. Readers not familiar with photosynthesis might find the increase in oxygen under non-aerated conditions nontrivial, but it is a well-established fact that dissolved oxygen levels rapidly rise in well illuminated microalgal cultures, which is a problem in large scale cultivation addressed with the use of costly equipment, such as degasser columns [29,30].

Dark or cold treatment, but not a combination of them, has also been shown to trigger de novo C20:5, n-3 biosynthesis in *N. oceanica* [31]. In this specific study, aeration was applied only at the control temperature setting (28–32 °C), while cultures in cold conditions (15 °C) were shaken manually twice per day (personal communication with corresponding author). Therefore, the observed positive effect of low temperature could be at least partially attributed to the same mechanism proposed above. The observed positive effect of darkness is also in agreement with our results, specifically with the negative effect of light period in PUFAs. The lack of positive interaction between low temperature and darkness further supports the idea that oxygen is responsible for the positive effect of decreasing temperature on PUFAs content, since in the absence of light there is no production of oxygen and thus only the positive effect of darkness remains, while increased oxygen solubility might also have a positive influence. These results are compatible with the observations of Harris and James, who were some of the first researchers to suggest that oxygen concentration directly affects fatty acid desaturation independently from temperature [32]. They observed an increase in FA desaturation in bulb tissue with either decreasing temperature or increased oxygen concentration, but the same was not observed in plant leaf tissue and *Chlorella vulgaris*. They also observed that in dark conditions increased oxygen concentrations enhanced FA desaturation also in leaf tissue and *C. vulgaris*. They concluded that the observed effect of low temperature in FA desaturation can be attributed to the increase in oxygen solubility. 

While the effect of temperature is straightforward, since decreasing temperature increases oxygen solubility in water, the effects of aeration depend on the net oxygen mass balance in the culture. Ronda et al. measured the dissolved oxygen concentration and gamma-linolenic acid (C18:3, n-6) biomass content of *Arthrospira platensis* in a not-well illuminated bubble column photobioreactor [33]. They observed an increase in dissolved oxygen concentration and C18:3, n-6 with increasing aeration rate. They attributed the positive effect of aeration to C18:3, n-6 to the increased dissolved oxygen. This demonstrates the complicated interplay of aeration, illumination, and biomass concentration or reactor geometry, since if the light availability is not adequate to support photosynthesis in a significant portion of the reactor, the net oxygen mass balance is negative in comparison to the equilibrium concentration, in which case increased aeration results in a higher dissolved oxygen concentration. The same is observed in heterotrophic production of PUFAs from microalgae and yeast, where the negative effects of oxygen depletion are well established [28,34,35]. In the context of the present study, our analysis suggests, probably for the first time in microalgal research, that under light replete conditions, a termination of aeration induces biosynthesis of PUFAs by the increase in intracellular oxygen.

Another way that oxygen could influence the fatty acid profile is the enzymatic or non-enzymatic oxidation of PUFAs to oxylipins, a class of molecules that act as mediators and have bioactive properties beneficial to human health [36]. Oxidative conditions such as those present under elevated oxygen concentration and saturating light intensity could enhance oxylipin synthesis, which could cause an observed reduction in the abundance of the respective fatty acid. While light intensity was negatively associated with PUFAs in the current study, the negative effect of the presence of aeration is not compatible with increased oxylipin synthesis, since an increase in PUFAs is observed under non-aerated conditions, which, as mentioned before, would lead to an increase in oxygen concentration. Regardless, the magnitude of the potential effect of oxylipins to the observed fatty acid profile and lipid concentration cannot yet be assessed due to the relative sparsity of information on the formation of oxylipins under different growth conditions in *Nannochloropsis oculata* or other microalgae. Future studies should focus on this very interesting class of molecules.

### 3.3. Cell Wall Stability Might Also Be Related to the Effects of Aeration

Another explanation for the negative effect of the presence of aeration on the content of PUFAs could be related to the shear stress caused by aeration. Polyunsaturated fatty acids are present in the cell membranes, which are attached to the cell wall via the cell cortex [37]. Calcium, which plays a role in cell wall stability in plants, had a significant negative effect of both C18:2 percentage in total FAs and its ratio to C16:0. Calcium starvation has been show to significantly increase the total lipid content of *Chlorella*, while the C18:2 percentage in total FAs decreased [38]. In freshwater microalgae, calcium can trigger homeoviscous adaptation by toughening the cell wall and leading to an increase in the cell membrane fluidity via accumulation of PUFAs [39]. The results presented here suggest that reduction of calcium concentration to levels lower than that of seawater (~0.01 M or 0.41 g L^−1^) might have a similar effect for marine microalgae, since the maximum calcium concentration in the current study was that of seawater. Additionally, calcium is important in the signaling of nitrogen starvation via its presence on membrane sensor proteins. Reduction of calcium concentration might limit the response to nutrient starvation and thus the accumulation of saturated FAs, and, in return, lead to an increase in the relative abundance of PUFAs [40]. 

Another parameter that could be related to the stability of the cell wall is silicon, which was significant for C16:1. Silicon is an important element for diatoms, which rely on it to create their frustules. In non-diatom microalgae like Ochrophyta, which contain the genus *Nannochloropsis*, silicon either is a component of structures in the cell or is accumulated in a non-localized manner [27]. Potassium is another critical nutrient for microalgae, playing a role in various cellular processes including enzyme activation, osmoregulation, and pH regulation. Potassium concentration and its interaction with phosphate had strong positive effect on C14:0/C16:0 and the C16:0 biomass content, respectively. Potassium nitrate increases lipid accumulation in comparison to sodium nitrate [41,42]. These results suggest the important roles of silicon and potassium in biofuels production.

### 3.4. Novel Effects of Magnesium and Manganese

Other micronutrients that were highlighted as significant in this study were magnesium and manganese, the former with a positive effect on C16:1, particularly via its notable negative interaction with the light flux per volume, and the latter with its strong positive effect on all parameters related to C18:2, especially via its interaction with temperature. Magnesium is a component of chlorophyll and thus one of the most critical micronutrients in photoautotrophic organisms. It is also important for ribosome stability and an activator of ribulose biphosphate carboxylase (RuBisCO) among other enzymes. Magnesium deprivation led to the highest monounsaturated FA content in *Auxenochlorella protothecoides* in one study [43]. The interaction of magnesium and light appears with a moderate severity index in a multivariable study of mixotrophic cultivation of algae isolated from a lentic system [44]. In the current study, the observed negative interaction of magnesium concentration with the light flux per volume could indicate that magnesium concentration upregulates chlorophyll production, which could lead to excessive light uptake under high light availability. Indeed at magnesium limited conditions increase in its concentration leads to increase in chlorophyll content [38]. However, the data used in this study did not include magnesium deprived conditions, since magnesium is abundant in sea water and added in adequate amounts in artificial sea water. Thus, the results of the current study might indicate a novel effect of magnesium at high concentrations. Manganese is an essential micronutrient for microalgae, as a component of PSII and enzyme cofactor, but is also considered a heavy metal. Manganese depletion can lead to an increase in polyunsaturated FAs in *Nannochloropsis oceanica* [9], which was not observed in the current study, despite the presence of manganese-depleted data points [45]. On the contrary, manganese had a (previously undocumented) strong positive effect on C18:2. Possible explanations are the upregulation of enzymes in the FA metabolism or upregulation of antioxidant enzymes like manganese superoxide dismutases [46], which could limit lipid peroxidation [28]. Lipid peroxidation increases with temperature, which, together with the strong positive interaction of manganese concentration with temperature, supports this idea [47]. 

### 3.5. Stressful Effects of Excessive Aeration Rate

An important parameter for saturated FAs and thus biodiesel production seems to be the aeration rate and its interplay with the partial CO_2_ pressure. While the presence of aeration is necessary for saturated FAs accumulation, excessive aeration rate seems to have a negative impact, which is alleviated with increasing CO_2_ partial pressure, as indicated by the positive interaction between aeration rate and the CO_2_ partial pressure. This is observed for both the C14:0 and C16:0 biomass contents. These results are supplementary to those of Spolaore et al. who studied the effect of aeration rate between 0.02 and 0.25 vvm, under ambient CO_2_ concentration, and reported the maximum of this range as the optimal value [48]. Additionally, those results agree with the data presented by Ajala and Alexander [11], who studied separately the effects of aeration and agitation on the proximate composition of *N. oculata* and other microalgae under a high CO_2_ partial pressure (2% or 0.02 atm at ambient pressure). Specifically, they presented the effects of shaking, aeration, and stirring, which indicate that at moderate and high aeration rates, the increase in agitation rate either by shaking or stirring initially increased the lipid content and after a certain threshold had a negative effect. On the other hand, a rise of aeration rate from 0.15 to 2 vvm increased the lipid content. These results might also be relevant to the negative effect of aeration on PUFA content, since, as discussed, an increase in saturated FAs will result in the decrease in the relative PUFA content. 

### 3.6. Nitrogen and Phosphate Stress Effects Agree with Well-Established Knowledge 

The effects of nitrogen and phosphate concentration were mostly in line with the existing literature, with nitrate being the most important N-source but also the most prevalent in the dataset. Interestingly, the negative effect of nitrogen concentration was more pronounced for C16:1 and C18:1 than for C16:0. In *Nannochloropsis* C16:0, C16:1, and C18:1 are the main components of TAGs [49,50], where they accumulate under stress, especially under nitrogen starvation, via de novo synthesis through the acyl-CoA-dependent pathway, or via conversion of membranes [51]. On the other hand, C18:2 and C20:5, n-3 are mainly present in polar lipids [50], and they are accumulated under conditions requiring membrane plasticity, such as low temperatures, while they are negatively affected by nutrient limitation. However, PUFAs might also be synthesized de novo during nutrient starvation and transferred to TAGs [51,52,53]. Such an effect was not observed in the final results presented in this article, since all variables for the biomass content of C20:5, n3 were excluded after the second round of LASSO regression, while nitrate or phosphate concentrations were not included in the model for C18:2 biomass content. 

However, the first round of LASSO (MinMSE) indicated a negative effect of all variables related to phosphate concentration on the biomass content in C20:5, n-3, which agrees with the findings of Matsui et al., who reported maximum accumulation of C20:5, n-3 at the initial stages of phosphate starvation [50]. Interestingly, in contrast to nitrate starvation, phosphate starvation seems to be more important for C16:0 than for C16:1 and C18:1, according to the final models. Shi et al. presented data showing a significant increase in C16:0 and C18:1 FA percentages after day 2 and day 4 of phosphate starvation, respectively, while no noticeable change was observed for C16:1 [54]. Interestingly, the 3-day average phosphate concentration was included in our models for C16:0 FA percentage and biomass content, while the 4-day average appeared in the models for C16:1 and C18:1. 

It could also be argued that the positive effect of biomass concentration on the biomass content in C18:2 might be related to nutrient starvation, since increasing biomass concentration will increase the consumption rate of resources. It seems to be more related, however, to the protective effects of cell shading under high light availability as it is indicated by the strong positive interaction with the light flux per volume, which has a negative coefficient in the model. 

In conclusion, the results presented agree with well-known facts of the FA profile and content of *N. oculata*, such as the effects of temperature and nutrient starvation, while less well-established effects reported for other *Nannochloropsis* species, such as those of potassium and manganese, were demonstrated for the first time in *N. oculata*. Novel findings include the effect of magnesium on C16:1/C16:0, the effect of calcium on C18:2, and the negative effect of the presence of aeration in PUFA content, which could be attributed to different mechanisms, possibly due to an increase in dissolved oxygen concentration, similar to that occurring under low temperatures.

## 4. Materials and Methods

A total of 121 observations were used in the analysis presented in this paper, out of which 30 came from novel experiments, 24 came from our previous work, and 67 came from other writers. The full dataset is provided in the Supplementary Materials (S1).

The next sections describe the growth conditions and analyses performed to the novel experiments, the methodology for data collection and processing, and, finally, the statistical methods used to evaluate the effects of different predictor variables to the fatty acid profile.

### 4.1. Chemicals and Reagents 

NaNO_3_ (PanReac, Barcelona, Spain) and NaH_2_PO_4_·2H_2_O (Honeywell, Tokyo, Japan) were used as nitrogen and phosphorus sources, respectively, while the trace elements Na_2_EDTA (Sigma, St. Luis, MO, USA), FeCl_3_·6H_2_O (Acros Organics, Geel, Belgium), CuSO_4_·5H_2_O (Sigma), ZnSO_4_·7H_2_O (Sigma), CoCl_2_·6H_2_O (Fisher Scientific, Waltham, MA, USA), MnCl_2_·4H_2_O (Acros Organics), and Na_2_MoO_4_·2H_2_O (Chem-Lab NV, Zedelgem, Belgium) were used for media preparation. Cyanocobalamin, Thiamine HCl, and Biotin were procured from Sigma-Aldrich. Chemicals used for analysis included ammonium bicarbonate (Sigma), HPLC-grade chloroform (Honeywell) and HPLC-grade methanol (Fisher Scientific).

### 4.2. Microalgal Species & Cultivation Conditions

*Nannochloropsis oculata*, was originally provided by the Laboratory of Zoology (Department of Biology, University of Patras, Greece) and was maintained as previously described [1,2]. Inoculum volume of 10 mL of the maintenance cultures was used to inoculate Erlenmeyer flasks of 500 mL capacity with 450 mL working volume. The medium used was four-times concentrated f/2 medium without silicate or vitamins. Initial pH was adjusted to 8 with addition of 1 Ν NaOH before the inoculation under a UV-sterilized laminar flow cabinet. Ambient air, filtered with a sterile 0.2 μm Whatman PTFE air filter, was provided at a rate of ~2.8 vvm, which also provided mixing, while light was supplied continuously from below at an intensity ~100 μmol m^−2^ s^−1^, measured at the illuminated surface, by 6000 K white LED light bulbs. After 15 days, these cultures were collected and used to inoculate new cultures at various conditions at an initial chlorophyll concentration of 5–6 mg Chl a L^−1^. Three sets of experiments were carried out. 

The first set aimed to study the effects of light intensity, aeration, and salinity. The duration of this experiment was 7 days, temperature was set to 20 °C, and other conditions were:

1. Aeration, 350 μmol m^−2^ s^−1^ and 60 ppt (mg L^−1^) salinity.
2. Aeration, 90 μmol m^−2^ s^−1^ and normal seawater salinity (~38 ppt).
3. Lack of aeration, 90 μmol m^−2^ s^−1^ and 60 ppt salinity.
4. Lack of aeration, 90 μmol m^−2^ s^−1^ and normal seawater salinity.

The same arrangement as that described for the pre-cultures was used, while the flasks subjected to non-aerated conditions were airtightly sealed with rubber stoppers. 

The second set of experiments also had the same arrangement as that used for pre-cultures, targeted the effects of temperature, initial pH, and light period, and was complementary to a published article of our group [1]. Each day, the pH was adjusted to a specific setpoint with HCl or NaOH. The duration of this experiment was 5 days, light intensity was fixed to ~100 μmol m^−2^ s^−1^, and the rest of the conditions were:

1. Initial pH 9.5, 20 °C, light period 12:12 (12 h light and 12 h dark).
2. Initial pH 6.5, 20 °C, light period 24:0 (continuous light).
3. Initial pH 8, 27.5 °C, light period 12:12.
4. Initial pH 8, 27.5 °C, light period 24:0.
5. Initial pH 6.5, 35 °C, light period 12:12.
6. Initial pH 9.5, 35 °C, light period 24:0.

The third experiment was a trial culture in a flat panel photobioreactor. Artificial sea water (ASW) with a brackish level of salinity enriched with 4 times concentrated f/2 medium [1] was used. The composition of the ASW (without the f/2 nutrients) was 2.71 g L^−1^ MgSO_4_·7H_2_O, 0.67 g L^−1^ KCl, 0.33 g L^−1^ CaCl_2_·2H_2_O and 16.67 g L^−1^ NaCl. The reactor was continuously illuminated from one side with a 4000 K LED panel, with the light intensity at the inner illuminated surface measured at 195 ± 28 μmol m^−2^ s^−1^, which corresponded to ~2249 μmol photons per second per m^3^ of working volume. Aeration with ambient air was provided at the bottom of the reactor with a horizontal sparger at a rate of ~0.33 vvm. Ambient temperature had little variation during the day and was measured daily, while pH was also monitored daily. The experiment lasted for 10 days, while biomass was collected for fatty acid profiling at days 5 and 7. Total Suspended Solids (TSS), nitrate concentration and total phosphorus concentration were also monitored daily as described below.

### 4.3. Analytical Measurements

Nitrate and total phosphorus concentration was measured daily at the filtered growth medium obtained from the TSS measurement. Nitrates were measured spectroscopically at 220 and 275 nm [55], while total phosphorus was measured as orthophosphates with the ascorbic acid method after hydrolyzation under low pH [55].

### 4.4. Biomass Composition Analysis

#### 4.4.1. Biomass Collection

At the end of each run, wet biomass was obtained via centrifugation at 3780× *g* for 7 min (Hermle, Z 366), washed with 0.5 M ammonium bicarbonate [56], freeze-dried (Telstar, LyoQuest), and stored in a desiccator. Ash content was then measured according to Standard Methods [55]. Subsequently, freeze-dried biomass was weighted and subjected to extraction and transesterification of FAs as described below.

#### 4.4.2. Fatty Acid Profiling and Quantification

Fatty acids were converted to their respective methyl esters (FAMEs) with the one-step in situ transesterification method of Indarti et al., as modified by Levine et al. [57,58], and subsequent analysis was made on a GC (Agilent Technologies, 7890A) equipped with a detector (FID) and a capillary column (DB–WAX, 10 m × 0.1 mm × 0.1 μm), as previously described [59]. To quantify the produced FAMEs, a reference standard (FAMQ-005, Accustandard) and an internal standard solution (C17: 0, Sigma) were used [59].

### 4.5. Choice of Regression Techniques and Data Preparation

The goal of the research presented in this paper was the selection of important variables for the fatty acid profile of *Nannochloropsis oculata* in a non-excluding manner. To that end, all of the available literature data was collected, evaluated, and combined with our own data. The end result was a dataset with a very large number of predictor variables, which increased further in number due to the introduction of dummy variables with the one-hot method in order to take into account categorical variables and fixed effects, as well as the use of 2-, 3-, and 4-day averages for some numerical variables, like pH, to include the history of growth conditions of the model [60]. Therefore, a regression method that can handle many predictors with a relatively small number of observations was necessary. LASSO regression is ideal for that case, since it is a method that eliminates non-important variables leading to sparse models while considering the mean squared error. To take into account interaction terms, a hybrid approach inspired by others was used [19]. In this hybrid technique, LASSO regression was used to evaluate the significance of variables or their interactions and reduce their number, while stepwise linear regression was used both to identify significant interaction terms and to provide the final refined model with confidence intervals for coefficients.

In the following paragraphs, the methodology used in this paper is presented, starting with the selection of predictor and response variables, followed by the handling of missing data, the data preparation steps, the hybrid regression, and the final model evaluation.

#### 4.5.1. One-Hot Encoding

One-hot encoding is a prevalent technique employed in the preprocessing of categorical data for machine learning algorithms. This method addresses the issue of categorical variables by transforming each category into a new binary feature, thereby enabling more effective handling by algorithms that require numerical input.

The one-hot encoding process operates by creating binary dummy variables for each category of the categorical variable. For a categorical variable with n categories, n − 1 binary features are created, each representing a single category. A given category is denoted by a ‘1’ in its corresponding binary feature and ‘0’ in all of the others. This transformation results in a binary vector for each category, where the vector length equals the number of categories in the original variable. To the n^th^ category, only zero values of the n - 1 dummy variables are assigned.

In the context of this study, one-hot encoding was utilized in two instances, one to account for the origin of the different datasets used, which were collected from various research groups, and the second to take into account the nitrogen source type (ammonium, nitrate or urea). Each research group or nitrogen source type was treated as a category within a categorical variable, and one-hot encoding was applied to this variable. This approach allowed for the preservation of crucial information about the data’s origin or nitrogen source type without imposing an arbitrary ordinal structure that could potentially bias the subsequent analysis. The resulting binary features were then incorporated into the data used for regression in subsequent steps, enabling a comprehensive evaluation of the influence of each research group on the response variable.

#### 4.5.2. Temporal Effects

For selected variables, for which the temporal profile was known or could be estimated, apart from the value at the day of biomass collection, 3 other variables were generated: the 2-, 3- and 4-day averages. In that way, a sense of evolution through time was incorporated into the analysis. The reasoning behind this step was that microalgae acclimate to environmental conditions, and each acclimation state might be influenced differently by environmental factors.

### 4.6. Handling of Missing Data

Handling missing data is a critical step in regression analysis, as the presence of missing values can lead to biased estimates, loss of efficiency, and complications in the model-building process. Various strategies exist for dealing with missing data, ranging from simple methods such as listwise deletion or mean imputation to more sophisticated techniques such as multiple imputation.

In the context of this study, missing data was addressed using multiple imputation, a statistically robust method that accounts for the uncertainty associated with missing values. Multiple imputation works by creating several different plausible imputed datasets and appropriately combining their results. This approach provides a more accurate estimate of the uncertainty due to missing data than single imputation methods, as it reflects the additional variance due to the imputation process.

The imputation process in this study was guided by model-based limits or educated assumptions. This means that the imputed values were not arbitrary but were based on logical assumptions about the relationships among variables or derived from statistical models built on the observed data. This approach ensures that the imputed values are plausible given the observed data and the assumptions made, thereby reducing the potential bias introduced by the imputation process. 

Regression was performed with each imputed dataset and the results were subsequently combined. Below the methodology used to select limits for the imputation of different missing data types is presented.

#### 4.6.1. Conversion of Cell and Optical Density to Biomass Concentration

Biomass concentration in terms of weight per volume of culture was used instead of optical density or cell count, since it is of higher practical importance when the microalgae production is considered and can be easily compared between different studies. Additionally, optical density can significantly vary with cultivation conditions and the state of the cell [61], while the same applies to the distribution of individual cell weight [62]. Due to interference of salt to the biomass concentration measurement in terms of dry weight (DW) [56], the ash-free dry weight (AFDW) was selected as a better alternative to DW [55]. 

In some cases, the biomass concentration was provided as a function of time, either in DW [8,63,64] or AFDW [1,2,65] basis. Original data presented in this article were in the AFDW basis. DW values were imputed as AFDW assuming 1% and 20% ash content for the upper and lower limits, respectively, while the AFDW values were repeated in each imputation dataset unchanged.

In one case, where optical density at 750 nm was provided along with the initial and final values of biomass concentration [7], the lower limit for biomass concentration was set by a linear relation between the initial and the final biomass concentration, while for the upper limit biomass, concentration was assumed to follow the same trend as the optical density. The initial and final biomass concentrations, used to calculate the time averaged biomass concentrations, were treated the same as described above, since they were provided by the authors.

In other cases, the cell density was provided as a function of time along with the final biomass concentration [6] or with information regarding the biomass productivity, which could be used to estimate the final biomass concentration [45,66]. In those cases, the initial biomass concentration was estimated using the cell count and the assumption that the average single cell weight was 10 pg for the lower limit and equal to the final biomass weight/cell count for the upper limit [67]. The temporal profile in both cases was assumed to be the same as the cell count temporal profile, with the lower case following a linear increase in cell weight from 10 pg to the final cell weight, and for the upper limit a constant cell weight was assumed.

The treatment of missing biomass concentration for the rest of the cases is explained in the Supplementary Materials (S4) [50,68,69].

#### 4.6.2. Estimation of Nitrogen and Phosphate Concentrations

In all cases, the initial nitrogen concentration was known, while some authors also provided the temporal evolution of nitrogen concentration [1,7,63,65] or provided the final values or information on the time when it was depleted [2,8,45]. In most cases, nitrate was the nitrogen source. Data of nitrate consumption and biomass production were used to fit a second order polynomial model consumption of nitrate vs. biomass concentration change with R squared 0.97. Predictor variables for the model were initial nitrate and biomass concentrations. The data indicate that higher initial nitrate and biomass concentrations lead to higher nitrate uptake, which has also been observed for other species [70], and which is compatible with nitrate reductase upregulation in the presence of high nitrate concentrations [71]. The highest dNO_3_dX value seems to be −0.0065 mg N-NO_3_ g^−1^ AFDW. The model is presented in the Supplementary Materials (S4). The limits for imputation of nitrate concentration were therefore estimated using the initial nitrate concentration and the upper/lower limits of biomass concentration for the lower and upper limit for nitrate concentration respectively. In cases where ammonia or urea were the nitrogen source, the concentration was either provided by the authors [45,69] or, in one case, assumed to follow the same trend as nitrate [64].

Data on phosphate availability was more sparse, with less authors providing information on the temporal profile or the final values of phosphate concentration [1,2,45,50,65]. The lower limit for imputation was estimated assuming biomass phosphorus content 2.5% and using the upper biomass concentration limit, while the upper limit was estimated assuming 0.5% biomass phosphorus content and using the lower biomas concentration limit. The limits of 0.5 and 2.5% were chosen based on a study on the luxury uptake of phosphate by *Nannochloropsis salina* [72]. 

#### 4.6.3. Estimation of pH and Inorganic Carbon

In this study, missing pH values were estimated using a two-step process: initial prediction and subsequent refinement. The equilibrium pH was initially approximated via the iPHREEQC COM module, which was interfaced with Matlab, utilizing the growth medium’s chemical composition and temperature as inputs. The PHREEQC library llnl.dat was used, since it is the most suitable for marine systems [73]. Due to expected discrepancies between the predicted and the actual pH values, residual modelling was implemented as a refinement step. This involved calculating residuals by subtracting 10^(- predicted equilibrium pH) from 10^(- real pH) in cases where real pH was known. These residual values were then used to create a linear model in Matlab, with predictors dN, dP (representing the total consumption of nitrate and phosphate at a given time in mol L^−1^, respectively) and the aeration rate in vvm. The resulting model demonstrated an R-squared value greater than 0.96, indicating a robust estimation of the deviation from the initial predictions, hence offering a more precise method for pH estimation. This model was utilized only in cases where ambient air was used for aeration. In cases where the CO_2_ concentration in the air feed exceeded that of ambient air, the pH was either provided by the authors or presumed to be the equilibrium value calculated with PHREEQC. The residual model is provided in the Supplementary Materials (S4), while information on the iPHREEQC COM module can be found on the USGS website.

Using the partial pressure of CO_2_ in the air feed and the medium composition and temperature, the concentration and speciation of inorganic carbon in the medium was also estimated using PHREEQC with the llnl.dat library, assuming equilibrium conditions. While at high biomass densities this might not be true, due to high carbon consumption [74], equilibrium conditions have been used to describe CO_2_ mass transfer in lab scale microalgal cultivation with success in the past [75]. For the novel data presented that involved non-aerated conditions, inorganic carbon was assumed to be zero after 7 days of cultivation due to the estimated carbon content of produced biomass. For the other case where no aeration was applied, the lower limit was also set to zero while the upper limit was that in equilibrium with air, calculated with PHREEQC as in the rest of the cases [66].

#### 4.6.4. Assumptions for Illumination Conditions

In all cases, the light intensity measured at the surface of the container used for cultivation was provided. The type of vessel as well as the working volume and orientation of the light source were also provided in most cases in the article or by the authors upon request. In one case, the type of reactor (cylindrical photobioreactor) was mentioned without any information on dimensions [63]. In that case, diameter was assumed to be between 5 and 10 cm, based on other examples of similar systems in the market and the literature. These boundaries were used to set the lower and upper limits for the illuminated surface, assuming illumination from one side of the reactor. Illumination related variables used for regression were the light intensity in μmol photons m^−2^ s^−1^ and the product of the light intensity with the illuminated surface divided by the working volume (μmol photons m^−3^ s^−1^), which was used as a measure of the light availability in the culture. All of the variables considered in this article are detailed in the Supplementary Materials (S1).

#### 4.6.5. Selection of Response Variables

Three types of response variables were considered: the percentage of a specific FA in total FAs, the ratio of that FA to C16:0 [76] that is usually the most abundant FA in *Nannochloropsis* [77], and the biomass content in the specific FA in terms of ash free dry weight [1].

### 4.7. Data Normalization

#### 4.7.1. Z-Score Normalization

Regularization methods like LASSO regression require normalization of the predictor variables to ensure that all predictors contribute equally to the model, regardless of their original scales. Without normalization, a predictor could dominate the model, not because it is necessarily more important or informative but due to its magnitude. This is particularly crucial for LASSO regression, which applies a penalty to the coefficients of the predictors in order to perform variable selection and prevent overfitting. The penalty is based on the magnitude of the coefficients, so if the predictors are not on the same scale, the penalty could unfairly affect predictors with larger scales. 

Z-score normalization is a good option for normalizing predictors because it not only brings all predictors onto the same scale (mean of 0 and standard deviation of 1) but also maintains the distribution and relationships in the data. This makes it easier for LASSO regression to fairly evaluate the importance of each predictor and make accurate predictions. The normalization of variables with the Z-score method has as follows:

Xi normalized = (Xi − MEANi)/SDi(1)

where:

Xi are the predictors;

MEANi are the mean values of all the observations for each predictor;

SDi are the standard deviation values for each predictor.

#### 4.7.2. Centering of Response Variables

Centering of response variables is an important step in regularization methods like LASSO and Ridge regression. The primary reason for this is to ensure that the intercept term of the model is not penalized. Regularization methods work by adding a penalty term to the loss function that the model seeks to minimize. This penalty term discourages the model from assigning too much importance to any one predictor, which can help prevent overfitting. However, the intercept term in a linear model is not a coefficient that multiplies a predictor variable but rather represents the expected value of the response variable when all of the predictors are zero. Penalizing the intercept term could therefore lead to a model that is biased and does not fit the data well. By centering the response variable (subtracting the mean from each value), we ensure that the intercept term represents the mean of the response variable, and we can safely apply the penalty to the other coefficients without affecting the intercept. This helps to maintain the interpretability of the model while still gaining the benefits of regularization. As already mentioned, centering is achieved simply by subtracting the mean value of the variable from each observation:

Y centered = Y − MEANY(2)

where:

Y is the response variable;

MEANY is the mean value of the response variable.

### 4.8. LASSO Regression

LASSO (Least Absolute Shrinkage and Selection Operator) regression is a method used in regression analysis and machine learning to perform variable selection and regularization. The goal of LASSO regression is to obtain the subset of predictors that minimizes prediction error for a quantitative response variable. The technique works by imposing a constraint on the model parameters that causes regression coefficients for some variables to shrink toward zero. Variables with a regression coefficient equal to zero after the shrinkage process are excluded from the model. This property of LASSO regression makes it particularly useful for analyzing datasets with many predictors.

The LASSO method minimizes the residual sum of squares subject to the sum of the absolute value of the coefficients being less than a tuning parameter, λ. Mathematically, this can be represented as:

minimize (1/(2 × n)) × Σ[Y − B_0_ − Σ[Bi × Xi]]^2^ + λ × Σ|Bi| (3)

where:

n is the number of observations;

B_0_ is the intercept term;

λ is a tuning parameter controlling the amount of shrinkage: the larger the value of λ, the greater the amount of shrinkage;

Bi are the parameter coefficients.

The first part of the equation, (1/2 × n) × Σ[Y – B_0_ − Σ[Bi × Xi]]^2^, is the mean squared error, a measure of the model’s prediction error. The second part, λ × Σ|Bi|, is the L1 penalty, which imposes a cost on the size of the coefficients.

In MATLAB, LASSO regression can be implemented using the “lasso” function. The function takes as input a matrix X of predictor variables and a vector Y of the response variable, and returns a vector of coefficients B. 

The minimization of the objective function in LASSO regression is typically achieved using a coordinate descent algorithm. This algorithm works by iteratively optimizing the objective function over one parameter at a time, holding all other parameters fixed. The algorithm continues until the change in the objective function is below a certain threshold, indicating that the solution has converged.

In the context of LASSO regression, the coordinate descent algorithm works by iteratively updating each regression coefficient Bi by minimizing the objective function with respect to Bi, holding all other coefficients fixed. This process is repeated until the coefficients converge to a solution.

The LASSO method has the advantage of producing simpler and interpretable models that involve only a subset of the predictors. However, the choice of the tuning parameter λ is crucial as it determines the level of penalty, and, hence, the number of predictors in the final model. Instead of using a single λ value, it is common practice to explore a range of λ values in order to identify the one that optimizes the model. Cross-validation is frequently employed in this process, with the optimal λ chosen as the one that minimizes prediction error. Another interpretation of optimal λ is the largest value of λ such that the mean cross-validated error is within one standard error of the minimum. This value of λ is often chosen for model selection because it provides a model that is simpler (i.e., has fewer predictors) but whose predictive performance is within one standard error of the best performing model. This is in line with the principle of parsimony, or Occam’s razor, which prefers simpler models when their performance is not significantly worse than more complex models.

### 4.9. Stepwise Regression with Selected Main Effects and Their Interactions

While LASSO regression is a powerful tool for parameter selection, it does not account for interactions between the different parameters examined. At the same time, interactions between the main effects might be of great importance and reveal novel aspects of the relationship between the studied variables. In the current article, a hybrid approach was followed to deal with these issues. Specifically, initially main effects were chosen with LASSO regression as described earlier, while a refinement step followed, during which the main effects chosen with LASSO and their interactions were fitted to a linear model using stepwise linear regression. This was performed in Matlab using the ‘‘stepwiselm’’ function and non-normalized data. Stepwise regression allows both for further variable selection with inclusion of significant interaction terms and comparison between selected variables via the *p*-value associated with each one. As in the case of LASSO regression, stepwise regression was also performed separately for each imputed dataset. In this case, however, the results were pooled using Rubin’s rule, since within-imputation variance existed. 

### 4.10. Rubin’s Rule

Rubin’s rule is a statistical method that is widely used in the analysis of multiple imputed datasets. The rule was proposed by Donald Rubin in 1987 and is designed to account for the uncertainty introduced by the imputation process when calculating estimates and their variances.

In the context of regression analysis, Rubin’s rule is used to combine estimates from multiple imputed datasets to produce a single estimate that reflects both the within-imputation variance and the between-imputation variance. The within-imputation variance is the average of the variances of the estimates from each imputed dataset, while the between-imputation variance is a measure of the variability of the estimates across the different imputed datasets.

The total variance, according to Rubin’s rule, is calculated as the sum of the within-imputation variance and a corrected form of the between-imputation variance. The correction factor accounts for the number of imputations. Specifically, the total variance (T) is calculated as follows:

T = W + (1 + 1/m) × B

where W is the within-imputation variance, B is the between-imputation variance, and m is the number of imputations.

Once the total variance is calculated, the total standard deviation can be derived by taking the square root of the total variance. The total standard deviation is a measure of the total uncertainty of the estimate, taking into account both the uncertainty within each imputed dataset and the uncertainty between different imputed datasets.

### 4.11. Post Hoc Analysis

In our study, we employed a multi-step approach to identify significant main effects and interaction terms, and to generate sparse, interpretable models. This process involved the use of LASSO regression, stepwise linear regression, and the application of trimmed mean calculations.

Initially, we utilized LASSO regression as a screening tool to identify important main effects. This method is known for its effectiveness in dealing with high-dimensional data and its ability to perform variable selection. We used three distinct criteria in the LASSO regression: the model that minimizes the mean squared error (MSE), the model that balances predictive accuracy and model simplicity (1SE), and a unique approach that we will refer to as “NoBlocking”.

The “NoBlocking” criterion involves examining the lambda value at which all dummy variables, accounting for the fixed effects of the data origin, are excluded from the model. The rationale behind this approach is that the exclusion of these blocking variables indicates that the original categorical variable, in that case the data origin, is not a significant predictor in the presence of the other variables in the model. This method should provide a means of identifying the most influential predictors in the model.

Following the LASSO regression, we performed stepwise linear regression using all variables selected with the three criteria. This step was crucial in selecting important interaction terms, which can often provide additional insights into the relationships between variables that are not evident when considering main effects alone.

We then conducted a second round of LASSO regression using all the main effects and interactions present in the models from the previous step. The purpose of this step was to further reduce the number of variables and assist in the generation of sparse models. LASSO’s ability to perform variable selection was again leveraged here to help simplify our models.

In each of these steps, we employed the use of trimmed mean calculations. For *p*-values, we focused on the mean rather than the trimmed mean. This is because a low mean *p*-value indicates the absence of large outliers and thus a narrower confidence interval, which is more significant. On the other hand, for variable coefficients, the trimmed mean was more important than the mean. This is because large outliers greater than zero can give the impression that a variable is significant, while in reality, it may have been excluded during LASSO regression.

Finally, we performed a second and final round of stepwise regression. For this step, we used the main effects that remained in the “NoBlocking” criterion, either individually or as part of an interaction, and the most important interactions, defined as those with a trimmed mean value of coefficients greater than zero. This final step resulted in sparse, interpretable models, providing us with a clear and concise understanding of the relationships between our variables.

## 5. Conclusions

The aim of this study was to investigate the available information on the fatty acid profile and content of *Nannochloropsis oculata* and identify key parameters for the abundance of selected fatty acids for this species. Novel data combined with information gathered from the literature was subjected to analysis using advanced regression methods and multiple imputation for missing data. This is probably the first time that LASSO regression has been used in the context of research on microalgal composition, and one of the first uses of a variation of the HDSI algorithm in general. Additionally, a novel approach to treat categorical variables in LASSO regression was introduced. The results agree with well-established facts regarding the effects of environmental conditions, such as temperature and pH, on the fatty acid content and profile of *N. oculata*, while novel observations were made, and the most important of these is the potential positive effect of the lack of aeration to the content in PUFAs, especially C20:5, n-3, the most valuable biomolecule produced by *Nannochloropsis*, with great importance to human nutrition and health. Additional highlights include the potentially significant (and previously undocumented for this species) effects of calcium, manganese, and magnesium. The research presented here paves the way for new experiments that will aim to investigate those effects, potentially contributing to the production of PUFAs and biofuels from *N. oculata* and other microalgal species.

## Figures and Tables

**Table 1:** Results of the 1^st^ round of LASSO regression for C14:0, C16:0, C16:1, C18:1, C18:2 and C20:5n3.

Variable *	C14:0	C16:0	C16:1	C18:1	C18:2	C20:5n3
cBOH3		−				+
cCa					−	
cCu				+		
cFe			+	−		
cK			−			
cMn			+	+	+	
cMg			+			
N-source NO3		+			−	
N-source Urea					+	
cNO3					+	
cNO3_2DaysAv				−		
cNO3_3DaysAv						+
cUrea_2DaysAv				−		
cUrea_4DaysAv					+	
cNa		−				
cNa_3DaysAv				+		
cPO4	+					
cPO4_4DaysAv					+	
cSO4						
cSi						+
T	+	+				−
T_2DaysAv						
T_3DaysAv			−			
T_4DaysAv					+	
Aeration		+	+	+	−	−
pH		+				
pH_4DaysAv		+				
CO2aq_4DaysAv						−
HCO3_2DaysAv			−			
LI		+				−
LV			−		+	
LV_3DaysAv				+		
LV_4DaysAv	−					
LP		+	+	+		−

* Positive signs indicate positive LASSO coefficients, whereas negative signs indicate negative LASSO coefficients.

**Table 2:** Results of the first round of LASSO regression for C14:0/C16:0, C16:1/C16:0, C18:1/C16:0, C18:2/C16:0, and C20:5n3/C16:0.

Variable *	C14:0/C16:0	C16:1/C16:0	C18:1/C16:0	C18:2/C16:0	C20:5n3/C16:0
cBOH3					+
cMn				+	
cMg		+			
cNO3_3DaysAv					+
cNa_4DaysAv					+
cPO4_4DaysAv				+	
cSi		+			+
S					+
T		+			−
Aeration					−
LI					−
LV		+			
LV_4DaysAv			+		
LP					−

* Positive signs indicate positive LASSO coefficients, whereas negative signs indicate negative LASSO coefficients.

**Table 3:** Results of the first round of LASSO regression for C14:0%AFDW, C16:0%AFDW, C16:1%AFDW, C18:1%AFDW, C18:2%AFDW, and C20:5n3%AFDW.

Variable *^,^**	C14:0%AFDW	C16:0%AFDW	C16:1%AFDW	C18:1%AFDW	C18:2%AFDW	C20:5n3%AFDW
cBOH3		−				+
cCo		−				
cCu					+	
cFe			+			
cK	+	+	+			
cMn					+	
N-source Urea					+	
cNO3_3DaysAv		−	+			
cNO3_4DaysAv				−		
cUrea_2DaysAv		−		−		
totalN_4DaysAv			+			
cPO4_4DaysAv		−	+	−		−
cSO4		−				
pCO2	+					
pCO2_2DaysAv				+		
pCO2_3DaysAv		+				
pCO2_4DaysAv	+		+			
T						−
T_3DaysAv		+				
Aeration		+	+	+		−
VVM						
VVM_2DaysAv	−	−				
pH_2DaysAv					−	
pH_4DaysAv			+			
CO2aq_2DaysAv				+		
CO2aq_4DaysAv	+	+	+			
LI	+	+	+	+		
LV_4DaysAv				+	+	
LP		+				
DW_2DaysAv					+	
DW_4DaysAv		+	+	+		

* Positive signs indicate positive LASSO coefficients, whereas negative signs indicate negative LASSO coefficients; ** AFDW: Ash Free Dry Weight.

**Table 4:** Stepwise regression results for the C14:0 fatty acid percentage, its ratio to C16:0 and its percentage in biomass in terms of AFDW.

C14:0			C14:0/C16:0			C14:0%AFDW		
	Estimate *	*p*-value **		Estimate *	*p*-Value **		Estimate *	*p*-Value **
(Intercept)	2.0 × 10^0^ ± 5.4 × 10^−1^	**0.028 ± 0.018** †	(Intercept)	1.4 × 10^−1^ ± 9.6 × 10^−2^	0.133 ± 0.271 †	(Intercept)	1.1 × 10^0^ ± 4.0 × 10^−2^	**0.000 ± 0.000** †
cPO4	8.5 × 10^2^ ± 3.6 × 10^3^	0.943 ± 0.231	cK	4.4 × 10^0^ ± 9.4 × 10^0^	0.811 ± 0.383	pCO2_4DaysAv	−4.1 × 10^0^ ± 1.1 × 10^0^	**0.030 ± 0.150** †
T	1.6 × 10^−1^ ± 4.6 × 10^−2^	**0.001 ± 0.002** †	cK:cPO4	9.6× 10^4^ ± 1.2 × 10^4^	**0.000 ± 0.000** †	VVM_2DaysAv	−7.2 × 10^−2^ ± 8.8 × 10^−3^	**0.000 ± 0.000** †
cPO4:T	5.6 × 10^2^ ± 2.4 × 10^2^	0.061 ± 0.230 †				CO2aq_4DaysAv	−6.2 × 10^0^ ± 4.6 × 10^1^	0.981 ± 0.136
						pCO2_4DaysAv:VVM_2DaysAv	2.2 × 10^1^ ± 3.4 × 10^0^	**0.000 ± 0.000** †
						pCO2_4DaysAv:CO2aq_4DaysAv	−1.1 × 10^1^ ± 2.8 × 10^2^	0.883 ± 0.317
*R*^2^ ***	0.32 ± 0.02		*R*^2^ ***	0.2 ± 0.03		*R*^2^ ***	0.52 ± 0.01	

* Pooled standard deviation (Rubin’s rule); ** Bold values indicate mean *p*-value value below 0.05, while the dagger symbol (†) indicates trimmed mean (80%) of *p*-value below 0.05; *** Adjusted values.

**Table 5:** Stepwise regression results for the C16:0 fatty acid percentage and its percentage in biomass in terms of AFDW.

C16:0			C16:0%AFDW		
	Estimate *	*p*-Value **		Estimate *	*p*-Value **
(Intercept)	−6.9 × 10^0^ ± 7.5 × 10^0^	0.197 ± 0.250	(Intercept)	−8.0 × 10^0^ ± 3.6 × 10^0^	0.118 ± 0.205 †
cK	1.0 × 10^3^ ± 1.2 × 10^3^	0.562 ± 0.494	cK	1.4 × 10^3^ ± 2.4 × 10^2^	**0.004 ± 0.058** †
NsourceNO3	3.2 × 10^0^ ± 3.5 × 10^0^	0.520 ± 0.496	NsourceNO3	2.0 × 10^−1^ ± 7.9 × 10^−1^	0.932 ± 0.247
cNO3_3DaysAv	8.5 × 10^1^ ± 5.8 × 10^2^	0.978 ± 0.146	cPO4_3DaysAv	−2.7 × 10^4^ ± 4.2 × 10^3^	**0.000 ± 0.000** †
cNa	−3.4 × 10^0^ ± 6.2 × 10^0^	0.749 ± 0.431	pCO2_3DaysAv	2.9 × 10^−2^ ± 1.6 × 10^0^	0.960 ± 0.194
cPO4_3DaysAv	−1.2 × 10^2^ ± 3.6 × 10^3^	0.990 ± 0.097	Aeration	1.8 × 10^−1^ ± 7.0 × 10^−1^	0.933 ± 0.245
Aeration	5.4 × 10^0^ ± 6.4 × 10^0^	0.553 ± 0.495	VVM_2DaysAv	−4.5 × 10^−1^ ± 7.7 × 10^−2^	**0.000 ± 0.000** †
VVM_2DaysAv	−6.0 × 10^−1^ ± 4.2 × 10^−1^	0.307 ± 0.461 †	pH	2.1 × 10^−2^ ± 1.7 × 10^−1^	0.984 ± 0.124
LI	−2.6 × 10^−4^ ± 2.3 × 10^−3^	0.987 ± 0.110	pH_4DaysAv	−1.5 × 10^−1^ ± 3.4 × 10^−1^	0.792 ± 0.399
LP	2.3 × 10^0^ ± 3.4 × 10^0^	0.673 ± 0.467	CO2aq_4DaysAv	3.6 × 10^1^ ± 1.9 × 10^2^	0.958 ± 0.200
DW_4DaysAv	−1.7 × 10^−2^ ± 1.8 × 10^−1^	0.990 ± 0.096	pCO2_3DaysAv:VVM_2DaysAv	1.5 × 10^1^ ± 6.9 × 100	0.057 ± 0.224 †
NsourceNO3:T_3DaysAv	5.1 × 10^−2^ ± 1.1 × 10^−1^	0.824 ± 0.380	Aeration:LI	2.0 × 10^−2^ ± 1.7 × 10^−3^	**0.000 ± 0.000** †
cNO3_3DaysAv:cNa	−3.2 × 10^3^ ± 2.4 × 10^3^	0.263 ± 0.440 †			
cNO3_3DaysAv:VVM_2DaysAv	−1.0 × 10^2^ ± 1.8 × 10^2^	0.737 ± 0.440			
T:pH	8.0 × 10^−2^ ± 1.1 × 10^−2^	**0.000 ± 0.000** †			
Aeration:LI	5.5 × 10^−3^ ± 9.3 × 10^−3^	0.717 ± 0.449			
Aeration:LP	5.3 × 10^0^ ± 4.6 × 10^0^	0.375 ± 0.483 †			
pH:LI	1.3 × 10^−4^ ± 4.6 × 10^−4^	0.922 ± 0.266			
*R*^2^ ***	0.51 ± 0.00		*R*^2^ ***	0.59 ± 0.01	

* Pooled standard deviation (Rubin’s rule); ** Bold values indicate mean *p*-value value below 0.05, while the dagger symbol (†) indicates trimmed mean (80%) of *p*-value below 0.05; *** Adjusted values.

**Table 6:** Stepwise regression results for the C16:1 fatty acid percentage, its ratio to C16:0, and its percentage in AFDW biomass.

C16:1			C16:1/C16:0			C16:1%AFDW		
	Estimate *	*p*-Value **		Estimate *	*p*-Value **		Estimate *	*p*-Value **
(Intercept)	2.8 × 10^1^ ± 4.1 × 10^−1^	**0.000 ± 0.000** †	(Intercept)	1.3 × 10^0^ ± 1.7 × 10^−1^	**0.000 ± 0.000** †	(Intercept)	2.4 × 10^1^ ± 3.8 × 10^0^	**0.000 ± 0.000** †
cNO3_3DaysAv	−2.8 × 10^0^ ± 1.6 × 10^2^	0.990 ± 0.098	cMn	2.6 × 10^4^ ± 2.5 × 10^4^	0.430 ± 0.488	cMg	−1.1 × 10^1^ ± 3.5 × 10^1^	0.907 ± 0.284
LV	−1.8 × 10^−6^ ± 3.5 × 10^−5^	0.997 ± 0.056	cMg	3.4 × 10^0^ ± 6.2 × 10^0^	0.767 ± 0.423	cNO3_3DaysAv	−5.9 × 10^3^ ± 1.9 × 10^3^	0.064 ± 0.243 †
cNO3_3DaysAv:LV	−5.9 × 10^−1^ ± 2.0 × 10^−1^	**0.008 ± 0.081** †	cNO3_3DaysAv	−1.7 × 10^1^ ± 3.9 × 10^1^	0.832 ± 0.372	cPO4_4DaysAv	−1.5 × 10^3^ ± 5.7 × 10^3^	0.937 ± 0.244
			totalN_4DaysAv	−5.1 × 10^0^ ± 1.2 × 10^1^	0.771 ± 0.415	cSi	−8.0 × 104 ± 5.5 × 10^4^	0.291 ± 0.435 †
			cPO4_4DaysAv	2.7 × 10^2^ ± 6.8 × 10^2^	0.861 ± 0.345	pH_4DaysAv	−1.8 × 10^0^ ± 3.1 × 10^−1^	**0.000 ± 0.000** †
			cSi	9.7 × 10^3^ ± 5.5 × 10^3^	0.233 ± 0.423 †	LI	−1.7 × 10^−2^ ± 5.7 × 10^−3^	0.063 ± 0.244 †
			pCO2_4DaysAv	6.1 × 10^−4^ ± 1.1 × 10^−2^	0.997 ± 0.055	DW_4DaysAv	−4.6 × 10^−1^ ± 4.5 × 10^−1^	0.483 ± 0.481
			T	−1.7 × 10^−3^ ± 1.3 × 10^−2^	0.964 ± 0.184	cNO3_3DaysAv:pH_4DaysAv	5.8 × 10^2^ ± 2.0 × 10^2^	0.069 ± 0.242 †
			T_3DaysAv	4.0 × 10^−4^ ± 1.1 × 10^−2^	0.948 ± 0.217	cSi:LI	8.2 × 10^2^ ± 1.6 × 10^2^	**0.000 ± 0.000** †
			Aeration	−8.2 × 10^−3^ ± 4.1 × 10^−2^	0.960 ± 0.195	pCO2_4DaysAv:DW_4DaysAv	2.3 × 10^0^ ± 5.2 × 10^−1^	**0.001 ± 0.001** †
			pH_4DaysAv	6.7 × 10^−4^ ± 5.3 × 10^−3^	0.984 ± 0.123			
			CO2aq_4DaysAv	−6.3 × 10^−1^ ± 4.9 × 10^0^	0.981 ± 0.136			
			HCO3_2DaysAv	2.5 × 10^−3^ ± 3.1 × 10^−2^	0.994 ± 0.078			
			cFe:cNO3_3DaysAv	6.7 × 10^5^ ± 1.6 × 10^6^	0.843 ± 0.363			
			cMg:LV	−4.3 × 10^−4^ ± 7.7 × 10^−5^	**0.000 ± 0.000** †			
			cNO3_3DaysAv:cPO4_4DaysAv	3.7 × 10^5^ ± 3.7 × 10^5^	0.449 ± 0.492			
			totalN_4DaysAv:HCO3_2DaysAv	−3.6 × 10^1^ ± 1.0 × 10^2^	0.880 ± 0.320			
			totalN_4DaysAv:LV	−2.4 × 10^−3^ ± 2.8 × 10^−3^	0.504 ± 0.489			
			T:Aeration	−5.1 × 10^−3^ ± 4.9 × 10^−3^	0.465 ± 0.497			
			T:pH_4DaysAv	−3.9 × 10^−3^ ± 5.5 × 10^−3^	0.318 ± 0.464 †			
			T_3DaysAv:pH_4DaysAv	7.4 × 10^−4^ ± 5.7 × 10^−3^	0.510 ± 0.496			
			Aeration:LP	−7.5 × 10^−2^ ± 9.0 × 10^−2^	0.578 ± 0.493			
*R*^2^ ***	0.09 ± 0.01		*R*^2^ ***	0.71 ± 0.00		*R*^2^ ***	0.56 ± 0.02	

* Pooled standard deviation (Rubin’s rule); ** Bold values indicate mean *p*-value value below 0.05, while the dagger symbol (†) indicates trimmed mean (80%) of *p*-value below 0.05; *** Adjusted values.

**Table 7:** Stepwise regression results for the C18:1 fatty acid percentage, its ratio to C16:0, and its percentage in biomass in terms of AFDW.

C18:1			C18:1/C16:0			C18:1%AFDW		
	Estimate *	*p*-Value **		Estimate *	*p*-Value **		Estimate *	*p*-Value **
(Intercept)	−2.1 × 10^0^ ± 1.4 × 10^0^	0.189 ± 0.115	(Intercept)	1.4 × 10^−2^ ± 5.9 × 10^−2^	0.509 ± 0.321	(Intercept)	2.7 × 10−1 ± 2.7 × 10−1	0.521 ± 0.129
cNO3_2DaysAv	−5.7 × 10^2^ ± 7.2 × 10^2^	0.464 ± 0.498	cNa_3DaysAv	1.6 × 10^−1^ ± 1.4 × 10^−1^	0.404 ± 0.480 †	cNO3_2DaysAv	−1.9 × 102 ± 1.9 × 102	0.507 ± 0.499
cNO3_4DaysAv	−5.0 × 10^2^ ± 6.7 × 10^2^	0.527 ± 0.499	CO2aq_2DaysAv	−3.2 × 10^−1^ ± 3.3 × 10^0^	0.990 ± 0.095	cNO3_4DaysAv	−1.8 × 102 ± 1.9 × 102	0.494 ± 0.499
cNa_3DaysAv	8.4 × 10^0^ ± 3.2 × 10^0^	**0.036 ± 0.179** †	cNO3_2DaysAv:LI	−1.8 × 10^−1^ ± 1.8 × 10^−1^	0.473 ± 0.499	pCO2_2DaysAv	8.1 × 10−2 ± 4.4 × 10−1	0.964 ± 0.184
cPO4_4DaysAv	5.1 × 10^1^ ± 9.8 × 10^2^	0.997 ± 0.055	cNO3_4DaysAv:LI	−1.5 × 10^−1^ ± 1.6 × 10^−1^	0.527 ± 0.499	Aeration	1.2 × 100 ± 2.9 × 10−1	**0.016 ± 0.006** †
pCO2_2DaysAv	6.0 × 10^0^ ± 1.6 × 10^0^	**0.000 ± 0.000** †	cNa_3DaysAv:LP	6.6 × 10^−2^ ± 1.0 × 10^−1^	0.685 ± 0.460	CO2aq_2DaysAv	−1.4 × 101 ± 6.4 × 101	0.954 ± 0.209
CO2aq_2DaysAv	−6.6 × 10^2^ ± 4.7 × 10^2^	0.192 ± 0.379 †	Aeration:LV_4DaysAv	1.3 × 10^−5^ ± 5.5 × 10^−7^	**0.000 ± 0.000** †	LI	4.1 × 10−4 ± 1.3 × 10−3	0.897 ± 0.303
LI	−2.3 × 10^−5^ ± 4.4 × 10^−4^	0.997 ± 0.057	Aeration:LP	1.4 × 10^−1^ ± 3.2 × 10^−2^	**0.005 ± 0.007** †	LV_4DaysAv	−2.3 × 10−5 ± 6.8 × 10−5	0.897 ± 0.304
LV_3DaysAv	−3.3 × 10^−5^ ± 1.9 × 10^−4^	0.967 ± 0.178				DW_4DaysAv	−1.0 × 10−3 ± 1.9 × 10−2	0.997 ± 0.055
DW_4DaysAv	1.0 × 10^−2^ ± 1.3 × 10^−1^	0.993 ± 0.080				pCO2_2DaysAv:LV_3DaysAv	5.9 × 10−4 ± 9.8 × 10−5	**0.012 ± 0.100** †
cNa_3DaysAv:LV_3DaysAv	1.0 × 10^−4^ ± 4.2 × 10^−4^	0.913 ± 0.281				LV_3DaysAv:DW_4DaysAv	2.0 × 10−5 ± 3.9 × 10−5	0.760 ± 0.427
Aeration:LV_4DaysAv	3.3 × 10^−4^ ± 1.0 × 10^−4^	0.087 ± 0.281 †				LV_4DaysAv:DW_4DaysAv	4.2 × 10−5 ± 2.4 × 10−5	0.240 ± 0.427 †
Aeration:LP	5.4 × 10^0^ ± 1.1 × 10^0^	**0.000 ± 0.000** †						
*R*^2^ ***	0.81 ± 0.00		*R*^2^ ***	0.74 ± 0.00		*R*^2^ ***	0.82 ± 0.00	

* Pooled standard deviation (Rubin’s rule); ** Bold values indicate mean *p*-value value below 0.05, while the dagger symbol (†) indicates trimmed mean (80%) of *p*-value below 0.05; *** Adjusted values.

**Table 8:** Stepwise regression results for the C18:2 fatty acid percentage, its ratio to C16:0 and its percentage in biomass in terms of AFDW.

C18:2			C18:2/C16:0			C18:2%AFDW		
	Estimate *	*p*−Value **		Estimate *	*p*-Value **		Estimate *	*p*-Value **
(Intercept)	1.6 × 10^1^ ± 7.6 × 10^−1^	**0.000 ± 0.000** †	(Intercept)	7.4 × 10^−1^ ± 4.1 × 10^−2^	**0.000 ± 0.000** †	(Intercept)	4.4 × 10^−1^ ± 1.3 × 10^−1^	0.191 ± 0.392 †
cCa	−9.3 × 10^2^ ± 6.1 × 10^1^	**0.000 ± 0.000** †	cCa	−4.1 × 10^1^ ± 3.4 × 10^0^	**0.000 ± 0.000** †	LV	−3.2 × 10^−5^ ± 1.7 × 10^−5^	0.887 ± 0.310
cPO4_4DaysAv	3.3 × 10^3^ ± 6.7 × 10^3^	0.795 ± 0.398	cMn	2.0 × 10^4^ ± 1.2 × 10^4^	0.270 ± 0.444 †	LV_4DaysAv	−2.9 × 10^−5^ ± 5.0 × 10^−5^	0.706 ± 0.453
Aeration	−4.5 × 10^0^ ± 3.8 × 10^−1^	**0.000 ± 0.000** †	cNO3	6.2 × 10^1^ ± 7.2 × 10^0^	**0.000 ± 0.000** †	DW_2DaysAv	1.4 × 10^−1^ ± 5.6 × 10^−2^	0.099 ± 0.284 †
cMn:T_4DaysAv	4.4 × 10^4^ ± 6.3 × 10^3^	**0.000 ± 0.000** †	cPO4_4DaysAv	1.2 × 10^2^ ± 3.6 × 10^2^	0.890 ± 0.313	cCa:pH_2DaysAv	−2.7 × 10^−2^ ± 4.8 × 10^−1^	0.997 ± 0.055
			Aeration	−1.8 × 10^−^3 ± 2.2 × 10^−2^	0.993 ± 0.081	cMn:T_4DaysAv	7.3 × 10^3^ ± 7.3 × 10^2^	**0.000 ± 0.000** †
			cMn:T_4DaysAv	1.7 × 10^2^ ± 3.9 × 10^2^	0.837 ± 0.370	cMn:DW_2DaysAv	2.5 × 10^3^ ± 1.0 × 10^4^	0.940 ± 0.232
			Aeration:pH_2DaysAv	−3.0 × 10^−2^ ± 3.2 × 10^−3^	**0.007 ± 0.081** †	LV:DW_2DaysAv	2.9 × 10^−5^ ± 8.2 × 10^−6^	**0.003 ± 0.058** †
						LV_4DaysAv:DW_2DaysAv	7.4 × 10^−6^ ± 1.5 × 10^−5^	0.810 ± 0.392
*R*^2^ ***	0.79 ± 0.00		*R*^2^ ***	0.72 ± 0.01		*R*^2^ ***	0.87 ± 0.02	

* Pooled standard deviation using Rubin’s rule; ** Bold values indicate mean *p*−value value equal or below 0.05, while the dagger symbol (†) indicates trimmed mean (80%) of *p*-value equal or below 0.05; *** Adjusted values.

**Table 9:** Stepwise regression results for the C20:5n-3 fatty acid percentage and its ratio to C16:0.

C20:5n3			C20:5n3/C16:0		
	Estimate *	*p*-Value **		Estimate *	*p*-Value **
(Intercept)	5.0 × 10^1^ ± 4.3 × 10^0^	**0.000 ± 0.000** †	(Intercept)	3.0 × 10^0^ ± 7.2 × 10^−1^	**0.000 ± 0.000** †
cNO3_3DaysAv	7.2 × 10^2^ ± 1.7 × 10^3^	0.843 ± 0.363	cBOH3	6.0 × 10^1^ ± 2.3 × 10^2^	0.936 ± 0.238
cNa_4DaysAv	−2.1 × 10^−1^ ± 3.9 × 10^0^	0.997 ± 0.058	cNO3_3DaysAv	−5.4 × 10^0^ ± 4.8 × 10^1^	0.987 ± 0.111
S	3.1 × 10^−3^ ± 5.8 × 10^−2^	0.997 ± 0.057	T	−5.4 × 10^−2^ ± 2.7 × 10^−2^	**0.008 ± 0.058** †
T	−7.4 × 10^−1^ ± 1.8 × 10^−1^	**0.001 ± 0.002** †	Aeration	−7.1 × 10^−1^ ± 1.9 × 10^−1^	**0.047 ± 0.211** †
Aeration	−1.1 × 10^1^ ± 3.5 × 10^0^	**0.003 ± 0.001** †	LP	−8.4 × 10^−1^ ± 8.1 × 10^−1^	0.268 ± 0.441 †
LP	−7.6 × 10^0^ ± 4.7 × 10^0^	0.127 ± 0.307 †	cNO3_3DaysAv:cNa_4DaysAv	−6.5 × 10^1^ ± 7.3 × 10^2^	0.369 ± 0.477 †
cNO3_3DaysAv:S	1.1 × 10^2^ ± 5.4 × 10^1^	0.157 ± 0.363 †	cNO3_3DaysAv:S	9.2 × 10^0^ ± 1.1 × 10^1^	0.410 ± 0.492
T:LP	−2.9 × 10^−2^ ± 1.4 × 10^−1^	0.892 ± 0.303	T:LP	7.0 × 10^−3^ ± 3.4 × 10^−2^	0.600 ± 0.480
Aeration:LI	−1.0 × 10^−2^ ± 8.0 × 10^−3^	0.233 ± 0.351	Aeration:LI	−2.1 × 10^−5^ ± 1.3 × 10^−4^	0.975 ± 0.151
			Aeration:LP	−3.2 × 10^−2^ ± 1.5 × 10^−1^	0.953 ± 0.211
*R*^2^ ***	0.42 ± 0.00		*R*^2^ ***	0.42 ± 0.01	

* Pooled standard deviation using Rubin’s rule; ** Bold values indicate mean *p*-value value equal or below 0.05, while the dagger symbol (†) indicates trimmed mean (80%) of *p*-value equal or below 0.05; *** Adjusted values.

## Data Availability

All data directly used to derive the results presented in this article is available in the Supplementary Materials. Additional data can be made available upon request.

## Supplementary Materials

The following supporting information can be downloaded at: https://www.mdpi.com/article/10.3390/md21090483/s1, Table S1: Variables and Data; Table S2: LASSO Regression Results; Table S3: Stepwise Regression Results, Table S4: Handling of Missing Data.
